# Supplementary material for: Individual-Level Heterogeneity in Mask Wearing during the COVID-19 Pandemic in Malaysia
Source: Am J Trop Med Hyg. 2021 Aug 9;105(6):1516–8. doi: 10.4269/ajtmh.21-0566 (PMC8641304; doi:10.4269/ajtmh.21-0566)
Supplement: Supplementary file 1 [file tpmd210566.SD1.docx]

**Supplemental Appendix: the scale of face mask-wearing behavior (In English/ Malay/Chinese)**

How frequent do you wear a face mask when you go outside of your house?/

*Berapa kerapkah anda memakai pelitup muka apabila keluar dari rumah anda?/*

当您要外出的时候，您佩戴口罩的频率为多少？

a) Never / *Tidak pernah pakai pelitup muka/* 从不佩戴

b) Rarely (less than 10% of the time) / *Jarang (kekerapan kurang daripada 10%)* /很少（不到10%的时间）

c) Occasionally (about 30% of the time) /*sekali-sekala (kekerapan lebih kurang 30%)* /偶尔（大约30%的时间）

d) Sometimes (about 50% of the time) /*Kadangkala (kekerapan lebih kurang 50%)/* 有时（大约50%的时间）

e) Frequently (about 70% of the time)/ *Selalu (kekerapan lebih kurang 70%)* /经常（大约70%的时间）

f) Usually (about 90% of the time) /*Kebiasaannya (kekerapan lebih kurang 90%)* /通常（大约90%的时间）

g) Every time /*Sentiasa pakai pelitup muka/*每次都佩戴
